# Comparison of Severity of Genitourinary Syndrome of Menopause Symptoms After Carbon Dioxide Laser vs Vaginal Estrogen Therapy: A Systematic Review and Meta-analysis

**DOI:** 10.1001/jamanetworkopen.2022.32563

**Published:** 2022-09-21

**Authors:** Yeu-Chai Jang, Chi Yan Leung, Hsi-Lan Huang

**Affiliations:** 1 Department of Obstetrics and Gynecology, Wan Fang Hospital, Taipei Medical University, Taipei City, Taiwan; 2 Department of Global Health Policy, Graduate School of Medicine, The University of Tokyo, Tokyo, Japan

## Abstract

**Importance:**

Vaginal estrogen for genitourinary syndrome of menopause (GSM) should be used with caution in women with contraindications, highlighting the need for effective treatment alternatives.

**Objective:**

To compare the severity of GSM after vaginal laser vs estrogen therapy.

**Data Sources:**

The PubMed, Embase, and Cochrane Library databases were searched for articles published from database inception to April 8, 2022, with no language restrictions. Reference lists were also searched.

**Study Selection:**

Randomized clinical trials (RCTs) that compared the use of lasers with vaginal estrogen in adults were selected.

**Data Extraction and Synthesis:**

Two investigators independently extracted data from included studies. The Cochrane risk of bias tool for RCTs was used to assess risk of bias of each study. A random-effects model was used to pool mean differences (MDs) with 95% CIs.

**Main Outcomes and Measures:**

Primary outcomes were Vaginal Analog Scale (VAS; higher scores indicate severer symptoms), Vaginal Health Index (VHI; higher scores indicate better vaginal health), Vaginal Maturation Index (VMI; higher scores indicate higher estrogen effect on the vaginal epithelium), Female Sexual Function Index (FSFI; higher scores indicate better female sexual function), and Sexual Quotient–Female (SQ-F; higher scores indicate better female sexual function) questionnaire scores. Urinary symptoms were assessed as an additional outcome. Data analyses were performed from April 9 to 12, 2022.

**Results:**

A total of 6 RCTs with 270 women with GSM were included (135 were randomized to laser therapy and 135 to estrogen therapy; mean age ranged from 54.6 to 61.0 years). No significant differences were found between carbon dioxide laser and vaginal estrogen from baseline to the end of follow-up in overall VAS scores (MD, −0.16; 95% CI, −0.67 to 0.36; *I^2^*, 33.31%), VHI (MD, 0.20; 95% CI, −0.56 to 0.97; *I^2^*, 83.25%), VMI (MD, −0.56; 95% CI, −1.14 to 0.02; *I^2^*, 35.07%), FSFI (MD, −0.04; 95% CI, −0.45 to 0.36; *I^2^*, 41.60%), and SQ-F (*P* = .37 based on 1 study). Other questionnaire-based outcome measures demonstrated no difference between groups from baseline to the end of follow-up for changes in urinary symptoms.

**Conclusions and Relevance:**

This systematic review and meta-analysis of RCTs found that vaginal laser treatment is associated with similar improvement in genitourinary symptoms as vaginal estrogen therapy. Further research is needed to test whether vaginal laser therapy could be a potential treatment option for women with contraindications to vaginal estrogen.

## Key Points

**Question:**

Are there differences in outcomes after carbon dioxide laser vs vaginal estrogen therapy in patients with genitourinary syndrome of menopause?

**Findings:**

This systematic review and meta-analysis of 270 women from 6 randomized clinical trials found that vaginal laser therapy, compared with vaginal estrogen, was associated with similar improvement in genitourinary syndrome of menopause.

**Meaning:**

Future noninferiority trials are needed to test whether vaginal laser therapy could be a potential treatment option for women with contraindications to vaginal estrogen.

## Teaser

This systematic review of women with symptoms of genitourinary syndrome of menopause compares symptom severity after vaginal laser vs estrogen therapy.

## Introduction

Genitourinary syndrome of menopause (GSM) is a highly prevalent condition, affecting 40% to 60% of postmenopausal women.^1^ The clinical symptoms and signs of GSM include vaginal burning, pruritus, dryness, dysuria, and dyspareunia.^2^ Genitourinary syndrome of menopause has been linked to estrogen deficiency, resulting in reduced elastin and collagen in vaginal tissue, thinning of vaginal epithelium, and an increase in vaginal pH.^2^ Prior literature has demonstrated that GSM was associated with poor quality of life and mental health.^3^ Of importance, these negative outcomes were observed among both sexually active and inactive women.^4^ In addition, the severity of untreated GSM is likely to increase over time.^2^

The first-line treatments for GSM are vaginal lubricants and moisturizers.^2^ Vaginal estrogen has been demonstrated to be effective in alleviating the symptoms of GSM.^5^ The mechanism of action includes a lower vaginal pH, an increased percentage of superficial cells with a lower percentage of parabasal cells, and a greater number of vaginal lactobacilli.^6,7^ However, the adherence rate ranged from only 52% to 74%.^8^ Of note, the evidence regarding the long-term effects of vaginal estrogen use on endometrial safety is currently limited.^2^ Vaginal laser therapy is a relatively new treatment, which creates microtrauma, promoting the thickening of epithelium, blood vessel formation, and collagen synthesis.^2,9^ Currently, because of the scarcity of available evidence, vaginal laser therapies are not recommended for treating the symptoms of GSM by the North American Menopause Society and the US Food and Drug Administration.^2,10^ A meta-analysis^11^ that incorporated 3 randomized clinical trials (RCTs) suggests that carbon dioxide laser therapy was superior to sham treatment in terms of satisfaction, Female Sexual Function Index (FSFI), Vaginal Analog Scale (VAS), and Urogenital Distress Inventory (UDI-6) scores. More recently, a meta-analysis^12^ that summarized data from 3 RCTs before 2020 reported that there was no clinical difference between energy-based treatments and hormonal therapy. Since then, 3 additional trials were published, but the results have not been systematically quantified in aggregate.^7,13,14^ In this systematic review and meta-analysis, we compared the severity of GSM among patients receiving carbon dioxide laser vs vaginal estrogen therapy.

## Methods

### Data Sources and Searches

We followed the Preferred Reporting Items for Systematic Reviews and Meta-analyses (PRISMA) reporting guideline for the reporting of the meta-analyses (eTable 1 in the Supplement).^15^ The protocol was registered in the International Prospective Register of Systematic Reviews (CRD42022322181). In this study, we systematically searched the PubMed, Embase, and Cochrane Library databases for articles published from database inception to April 8, 2022, with no language restrictions (details of search strategies are described in eTables 2-4 in the Supplement). A manual screening of reference lists of relevant included articles and reviews was conducted to supplement the search.

### Study Selection

**Figure 1.**
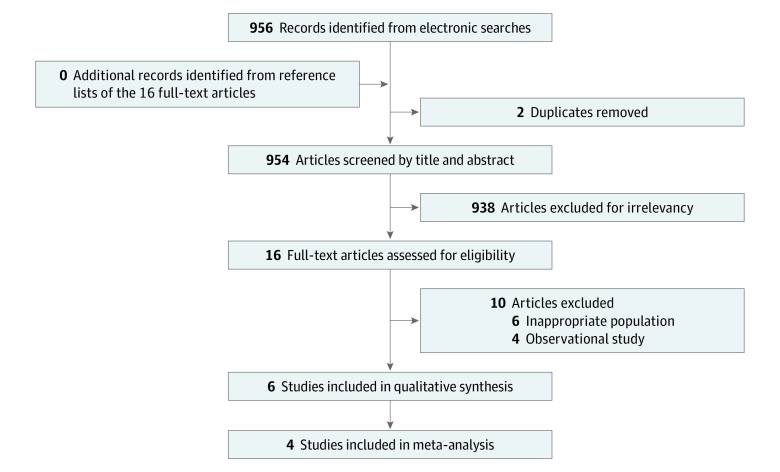
Study Selection

Two reviewers (C.Y.L. and H.L.H.) independently searched the title and abstract for potentially eligible RCTs comparing the efficacy of vaginal laser therapy and vaginal estrogen therapy in women with GSM according to prespecified methodologic criteria: (1) published original articles of RCTs, (2) studies that enrolled women, and (3) vaginal laser therapy and vaginal estrogen therapy as the interventions of interest. Disagreement on eligibility was resolved by discussion with a third reviewer (Y.C.J.). We identified 954 potentially relevant studies after initial literature search and exclusion of duplicated studies (literature search details and process are presented in Figure 1). Of the 16 studies that underwent full-text review, 10 were excluded because 6 of them evaluated irrelevant populations and 4 were observational studies.

### Outcome Measures

Primary outcomes included VAS, Vaginal Health Index (VHI), Vaginal Maturation Index (VMI), FSFI, and Sexual Quotient–Female (SQ-F) questionnaire scores. Urinary symptoms were assessed as an additional outcome using UDI-6, International Consultation on Incontinence Questionnaire–Short Form (ICIQ-UI SF), and International Consultation on Incontinence Questionnaire Overactive Bladder (ICIQ-OAB) scores. The VAS is a validated instrument with a continuous scale designed to capture the severity of vaginal symptoms (dyspareunia, dysuria, vaginal dryness, burning, and itching), in which 0 represents no symptoms and 10 represents the worst possible symptoms.^16^ The VHI, with scores ranging from 5 to 25, consists of 5 parameters (vaginal elasticity, epithelial integrity, fluid volume, moisture, and PH).^17^ A VHI score of 25 suggests no clinical signs of GSM. The percentage of parabasal, intermediate, and superficial cells from vaginal cytologic samples that contain at least 100 smear cells is used to obtain the VMI, ranging from 0 to 100, with a higher value indicating a higher estrogen effect on the vaginal epithelium.^18^ The FSFI is a 19-item symptom inventory developed to evaluate 6 domains of female sexual function (desire, arousal, lubrication, orgasm, satisfaction, and pain), ranging from 2 (severe dysfunction) to 36 (no dysfunction).^19^ The SQ-F questionnaire is a validated instrument that consists of 10 questions. The overall score ranges from 0 to 100, with higher scores suggesting better sexual satisfaction and performance.^20^ The UDI-6 is a 6-item symptom inventory used to assess the impact of urinary incontinence on quality of life, with an overall score of 0 to 100.^21^ Higher scores on the UDI-6 indicate greater disability.^22^ The ICIQ-UI SF and ICIQ-OAB are patient-administered questionnaires used to evaluate the frequency and severity of urinary symptoms, the occurrence of overactive bladder, and their impact on quality of life, with higher overall scores indicating greater symptom severity.^23^

### Data Extraction and Quality Assessment

Using a standardized prespecified data extraction form, 2 reviewers (C.Y.L. and H.L.H.) independently extracted the following data from identified articles: name of first author, publication year, country, study design, number and characteristics of participants, interventions, outcome measures, and results. A third reviewer (Y.C.J.) crosschecked the abstracted data for accuracy. Two reviewers (C.Y.L. and H.L.H.) applied the revised Cochrane risk of bias tool for RCTs to independently assess the risk of bias of each study. Disagreements were resolved by discussion between reviewers.

### Statistical Analysis

In the analysis, pooled mean difference (MD) of change in VAS, VHI, VMI, and FSFI scores between vaginal laser therapy and topical estrogen and corresponding 95% CIs were calculated using random-effects meta-analysis with restricted maximum likelihood method.^24,25^ We also performed random-effects meta-analysis to estimate the difference in mean change from baseline to the end of follow-up for the laser group and the estrogen group. We assessed the statistical heterogeneity using *I^2^* statistics. The cut-off values for heterogeneity were defined as 25% for low heterogeneity, 50% for moderate heterogeneity, and 75% for high heterogeneity.^26^ In this study, a 2-sided *P* < .05 was considered statistically significant. Statistical analyses were performed from April 9 to 12, 2022, using Stata MP, version 16.1 (StataCorp LLC).

## Results

### Literature Search

Six RCTs were included in this review, of which 3 provided data on VHI and FSFI,^9,13,27^ 2 on VAS and VMI,^9,13^ 1 on SQ-F,^7^ and 1 on urinary symptoms (ICIQ-UI SF and ICIQ-OAB).^14^ Therefore, 6 RCTs were included in qualitative synthesis and 4 in the meta-analysis. No additional study was identified after reviewing the reference lists of eligible articles. Details on excluded studies are given in eTable 5 in the Supplement.

### Trial Identification and Risk of Bias

**Figure 2.**
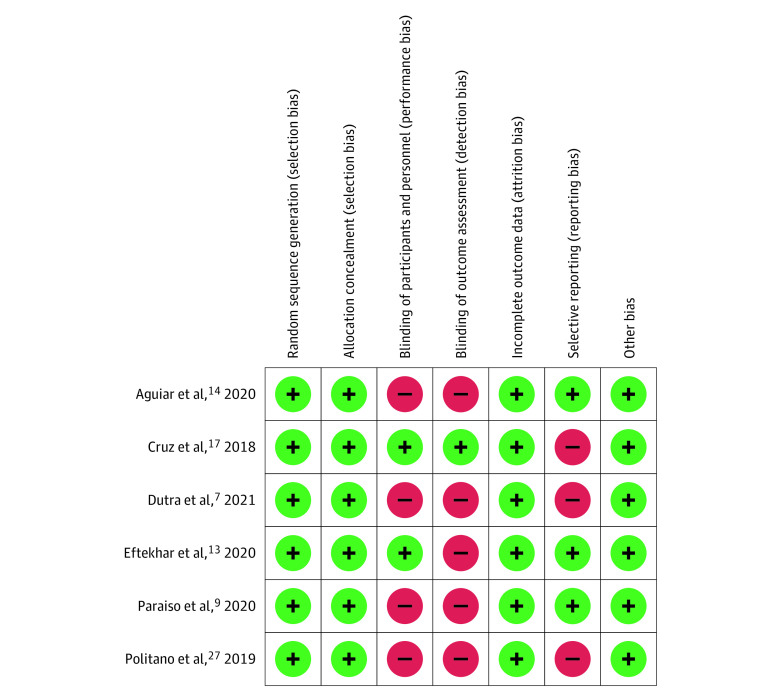
Risk of Bias Summary Green indicates low risk of bias; red indicates high risk of bias.

The included studies had a total of 270 participants (135 were randomized to laser therapy and 135 to estrogen therapy), with a mean age ranging from 54.6 to 61.0 years. The studies were published between 2018 and 2021. Enrolled patients were from Brazil, Iran, and the US. Baseline characteristics of the included trials are presented in the Table. The risk of bias of each trial across 5 domains evaluated is shown in Figure 2. Overall, risk of selection and attrition biases was low. Three studies had a high risk of reporting biases. We judged most of the trials as having high risk of performance and detection biases because of unblinding and open-label designs.

**Table.** Baseline Characteristics of Clinical Trials

Source	Study design	Study period	Participants, No.		Age, mean, y	Follow-up period, mo	Outcomes	Therapeutic protocol	
			Laser group	Estrogen group				For vaginal laser therapy	For vaginal estrogen
Dutra et al,^7^ 2021, Brazil	Controlled, unblended randomized clinical trial	Feb 2017-Feb 2018	13	12	55.3	4	VMI, Breslow thickness of mucosa, SQ-F	Fractional carbon dioxide laser system (power, 30 W; dwell time, 1000 μs; smart stack, 2), 3 sessions (1/mo)	1 mg of estriol cream daily for 30 d, followed by twice weekly for 2 mo
Paraiso et al,^9^ 2020, US	Multicentered, single-blinded randomized clinical trial	Jun 2016-Sep 2017	34	35	61	6	VAS, FSFI, UDI-6, VHI, VMI	Fractional microablative carbon dioxide laser system (power, 30 W; dwell time, 1000 μs; smartstack, 1 at the first session and 3 at the other 2 sessions), 3 sessions (once at least 6 wk apart)	0.5 g of conjugated estrogens vaginal cream daily for 14 d, followed by 0.5 g twice weekly for 24 wk
Eftekhar et al,^13^ 2020, Iran	Controlled randomized clinical trial	Nov 2017-Jan 2018	25	25	Estrogen: 57; laser: 54.6	6 (3 treatment +3 follow-up after treatment)	FSFI, VHI	Fractional carbon dioxide laser system (power, 40 W; dwell time, 1000 μs; smartstack 1 at the first session and 3 at the third session), 3 sessions (1 per month)	0.625 mg of conjugated estrogens vaginal cream was used for a third of the applicator 3 times weekly for 3 mo
Aguiar et al,^14^ 2020, Brazil	Randomized clinical trial	Mar 2017-Nov 2018	24	24	57.28	3.5 (14 wk)	ICIQ-UI SF, ICIQ-OAB	Fractional carbon dioxide laser system (power, 40 W; dwell time, 1000 μs; smart stack, 2 for applicator 360° and 3 for applicator 90° single-angle “closed” probe), 3 sessions (1 session 30-45 d apart)	10 mg of vaginal promestriene 3 times per week for 3 mo
Cruz et al,^17^ 2018, Brazil	Double-blinded, controlled randomized clinical trial	Jan 2015-May 2015	15	15	Estrogen: 56.9; laser: 55.9	5 (20 wk)	VHI, VAS, FSFI, VMI	Fractional microablative carbon dioxide laser system (power, 30 W; dwell time, 1000 μs; smart stack, 2), 2 sessions (1 session every 4 wk)	1 mg of vaginal estriol 3 times weekly for 20 wk
Politano et al,^27^ 2019, Brazil	Controlled randomized clinical trial	Mar 2017-Nov 2018	24	24	Estrogen: 57.21; laser: 57.83	3.5 (14 wk)	VHI, FSFI	Fractional carbon dioxide laser system (power, 40 W; dwell time, 1000 μs; smart stack, 2), 3 sessions (1 per month)	One vaginal applicator containing 1 g of cream and 10 mg of promestriene 3 times weekly for 12 wk

Abbreviations: FSFI, Female Sexual Function Index; ICIQ-OAB, International Consultation on Incontinence Questionnaire Overactive Bladder; ICIQ-UI SF, International Consultation on Incontinence Questionnaire–Short Form; SQ-F, Sexual Quotient–Female questionnaire; UDI-6, Urogenital Distress Inventory; VAS, Vaginal Analog Scale; VHI, Vaginal Health Index; VMI, Vaginal Maturation Index.

### Outcomes

**Figure 3.**
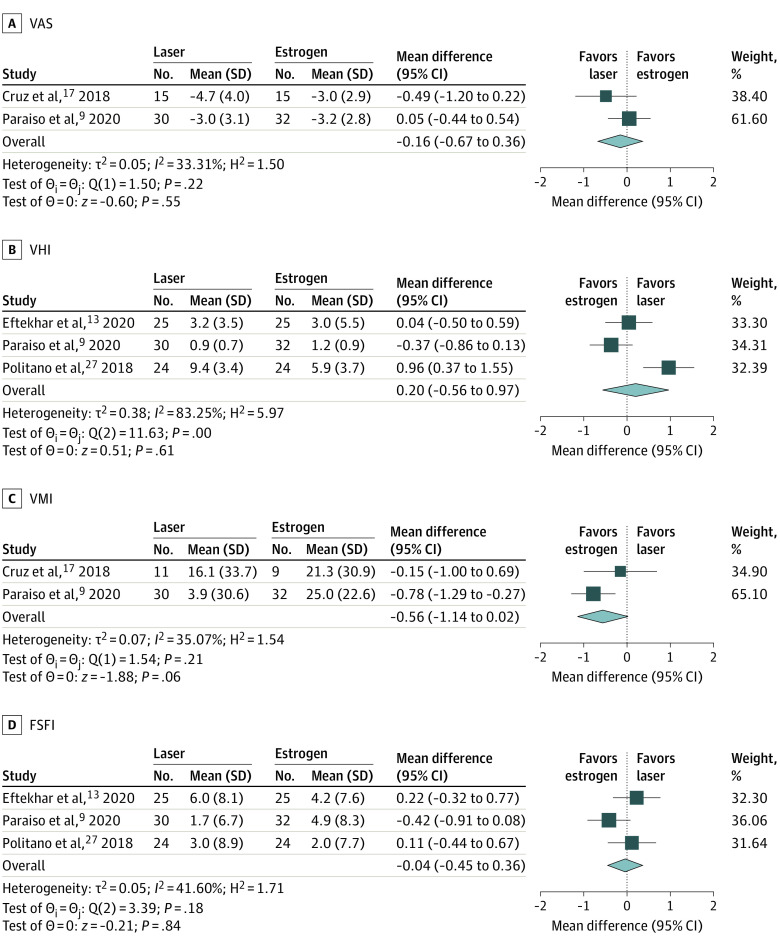
Mean Difference in Genitourinary Syndrome of Menopause Scores Between Laser-Treated and Estrogen-Treated Groups Mean difference in Vaginal Analog Scale (VAS) (A), Vaginal Health Index (VHI) (B), Vaginal Maturation Index (VMI), and Female Sexual Function Index (FSFI) scores between laser-treated and estrogen-treated groups.

In the analysis of VAS, no significant difference was found between carbon dioxide laser and vaginal estrogen treatment from baseline to the end of follow-up in overall VAS scores. Calculation using the random-effects model estimated a between-group MD of −0.16 (95% CI, −0.67 to 0.36; *I^2^*, 33.31%) (Figure 3A), with a statistically significant MD from baseline to the end of follow up of −3.64 (95% CI, −5.28 to −2.01) in the laser group and −3.09 (95% CI, −3.90 to −2.28) in the estrogen group (eFigures 1-2 in the Supplement). Three trials reported VHI scores comparing laser and estrogen therapy. These VHI scores did not significantly differ between the laser group and the vaginal estrogen group (4.46 vs 3.30; MD, 0.20; 95% CI, −0.56 to 0.97; *I^2^*, 83.25%) (Figure 3B; eFigures 3-4 in the Supplement). Analysis of VMI data comparing laser and estrogen therapy revealed no significant difference between groups from baseline to the end of follow-up (MD, −0.56; 95% CI, −1.14 to 0.02; *I^2^*, 35.07%) (Figure 3C). A significant difference was found in VMI scores in the estrogen group (MD, 24.52; 95% CI, 17.22-31.82) but not in the laser group (MD, 7.04; 95% CI, −3.41 to 17.49) after the end of treatment (eFigures 5-6 in the Supplement). In addition, Dutra et al^7^ reported that participants who received vaginal estrogen had a nonsignificant trend of higher VMI than the laser group (*P* = .073). Comparing the laser group with the estrogen group, the MD in the pooled analysis for FSFI did not differ significantly between carbon dioxide laser therapy and vaginal estrogen from baseline to the end of follow-up (MD, −0.04; 95% CI, −0.45 to 0.36; *I^2^*, 41.60%), with a MD in the FSFI score of 3.42 (95% CI, 0.82-6.02) in the laser-treated patients vs 3.77 (95% CI, 2.06-5.49) in the estrogen-treated patients (Figure 3D; eFigures 7-8 in the Supplement). The Brazilian SQ-F questionnaire used in 1 trial to assess sexual function before and after treatments showed that both the laser (*P* < .001) and estrogen group (*P* < .001) had significant improvement and there was no difference between groups (*P* = .37).^7^

Urinary symptoms assessments (UDI-6, ICIQ-UI SF, and ICIQ-OAB) were reported in 2 studies.^9,14^ One of these trials provided evidence for UDI-6 from baseline to 6 months in the laser and estrogen groups (−9.4 vs −6.2; MD, −0.23; 95% CI, −0.72 to 0.27) (eFigure 9 in the Supplement).^9^ The other trial reported no difference between the laser and estrogen groups from baseline to the end of follow-up in the change in the total scores for ICIQ-UI SF (−3.14 vs −1.53; MD, −0.29; 95% CI, −0.85 to 0.27) and ICIQ-OAB (−0.91 vs −1.16; MD, 0.08; 95% CI, −0.48 to 0.64) (eFigures 10-11 in the Supplement).^14^

## Discussion

In this systematic review and meta-analysis of 270 women from 6 RCTs, vaginal laser therapy vs vaginal estrogen treatment had similar improvement in VAS, VHI, VMI, and FSFI scores. Although the symptoms of GSM were negatively associated with quality of life and mental health,^3^ the Women’s EMPOWER survey suggested that only 50% of women with GSM sought medical help.^28^ The major concerns about initiating vaginal estrogen therapy include adverse effects, the safety of long-term use, and cancer risks.^29^ Among our included studies, Paraiso et al^9^ reported 1 case of breast tenderness, 1 case of migraine, 1 case of abdominal cramping, and 2 cases of vaginal bleeding among 32 participants in the vaginal estrogen group. No adverse event was observed in the studies by Dutra et al,^7^ Eftekhar et al,^13^ Aguiar et al,^14^ Cruz et al,^17^ and Politano et al.^27^ The endometrial safety of vaginal estrogen has been investigated in RCTs, which showed that women using vaginal estrogen had similar rates of endometrial cancer and endometrial hyperplasia to the general population.^30^ The longest follow-up among those RCTs was 52 weeks, however.^30^ In the Women’s Health Initiative study,^31^ a prospective cohort study of 45 663 women with a median follow-up of 7.2 years, the risks of breast cancer, colorectal cancer, and endometrial cancer were similar between users of vaginal estrogen and nonusers. Nevertheless, vaginal estrogen is contraindicated in women with undiagnosed vaginal bleeding and should be administered with caution in women with estrogen-dependent cancers,^2^ suggesting the importance for exploring alternative treatment.

A previous meta-analysis^32^ of observational studies suggested laser therapy is likely to be effective in improvement of GSM, which was in accordance with our meta-analysis of 6 RCTs with relatively consistent study designs. In our analysis, all included studies applied the laser regimen of 2 to 3 sessions with an interval of 4 to 6 weeks.^7,9,13,14,17,27^ There is no guideline regarding the optimal number of sessions. A prospective pilot study^33^ of 53 women reported that the intensity of vaginal dryness and dyspareunia was further reduced after the fourth and fifth sessions of laser therapy. However, the safety of vaginal laser treatment and adverse events associated with multiple sessions of laser therapy have yet to be determined. Although vaginal laser therapy has been proposed to be a potential treatment option for women who have contraindications to vaginal estrogen,^17^ it is important to note that in all included trials, women with contraindications to hormonal treatment were excluded from the study population. Direct comparison between laser therapy and topical estrogen among women with contraindications for hormonal therapy would be unethical. Our finding that there is no difference in treatment outcomes between vaginal laser and topical estrogen treatments does not imply that women with GSM and contraindications to estrogen therapy should undergo laser therapy. There may be concerns that laser therapy is an invasive procedure with the potential for complications, such as vaginal laceration, scarring, and perforation. Further research into the clinical benefits and harms of laser therapy use in women with contraindications to hormonal treatment is warranted.

To our knowledge, the current study is the most recent to systematically quantify RCTs comparing the treatment outcomes of vaginal laser vs vaginal estrogen therapy. Our study included both objective (VHI and VMI) and subjective (VAS and FSFI) measures to comprehensively inform the differences between vaginal estrogen and vaginal laser therapy.

### Limitations

This study has several limitations. First, the included studies followed up patients for up to 6 months,^7,9,13,14,17,27^ which renders the interpretation for long-term outcomes and safety difficult. Future studies to examine improvement in GSM beyond 12 months are warranted. Second, although other outcomes in the current study showed moderate heterogeneity, the high heterogeneity in the outcome for VHI limits the interpretability of our estimates. The number of included studies did not allow us to perform subgroup analyses. In addition, we were unable to assess publication bias because of the number of included studies. Third, it is possible that the posttreatment care received by patients in each group varied because most of the included studies were not blinded. Fourth, VMI scores in the study by Paraiso et al^9^ were only available for 55% (34 of 62) of participants. Although the *P* value in the study remained statistically significant after adjusting for age, menopause status, prior use of estrogen, and sexual activity, residual confounding cannot be ruled out. Fifth, reporting of outcome data was inconsistent in the included trials; therefore, our results may be subject to selective reporting bias. Sixth, our results need to be interpreted with caution because noninferiority and equivalence were not tested in the current analysis. The non–statistically significant difference demonstrated in the current study does not necessarily suggest that laser therapy is equivalent or not inferior to topical estrogen therapy. Further noninferiority trials comparing vaginal laser and estrogen therapy are warranted to clarify our findings. Seventh, given the absence of individual-level data, we cannot evaluate the baseline characteristics and facilitate standardized analyses across studies.

## Conclusions

This systematic review and meta-analysis found that vaginal laser treatment had similar improvement to vaginal estrogen therapy in terms of VAS, VHI, VMI, and FSFI scores. These findings offer important insight into alternative treatment options for women with GSM and contraindication to hormonal therapy. Future studies with adequate statistical power and sufficient follow-up are warranted.

## Notes

Supplement. **eTable 1.** Preferred Reporting Items for Systematic Reviews and Meta-Analyses (PRISMA) Guidelines for the Reporting of Meta-analyses **eTable 2.** Search Strategies on the PubMed **eTable 3.** Search Strategies on the Ovid Embase **eTable 4.** Search Strategies on the Cochrane Library **eTable 5.** List of References With Final Exclusion Reasons **eFigure 1.** Mean Difference of Improvement in VAS From Baseline to the End of Follow-up in the Laser Group **eFigure 2.** Mean Difference of Improvement in VAS From Baseline to the End of Follow-up in the Estrogen Group **eFigure 3.** Mean Difference of VHI From Baseline to the End of Follow-up in the Laser Group **eFigure 4.** Mean Difference of VHI From Baseline to the End of Follow-up in the Estrogen Group **eFigure 5.** Mean Difference of VMI From Baseline to the End of Follow-up in the Laser Group **eFigure 6.** Mean Difference of VMI From Baseline to the End of Follow-up in the Estrogen Group **eFigure 7.** Mean Difference of FSFI From Baseline to the End of Follow-up in the Laser Group **eFigure 8.** Mean Difference of FSFI From Baseline to the End of Follow-up in the Estrogen Group **eFigure 9.** Mean Difference of UDI-6 From Baseline to 6 Months in the Laser and Estrogen Group **eFigure 10.** Mean Difference Between the Laser and Estrogen Groups From Baseline to the End of Follow-up for ICIQ-UI SF **eFigure 11.** Mean Difference Between the Laser and Estrogen Groups From Baseline to the End of Follow-up for ICIQ-OAB
