# Supplementary material for: Comparison of Severity of Genitourinary Syndrome of Menopause Symptoms After Carbon Dioxide Laser vs Vaginal Estrogen Therapy: A Systematic Review and Meta-analysis
Source: JAMA Netw Open. 2022 Sep 21;5(9):e2232563. doi: 10.1001/jamanetworkopen.2022.32563 (PMC9494191; doi:10.1001/jamanetworkopen.2022.32563)
Supplement: Supplement. — eTable 1. Preferred Reporting Items for Systematic Reviews and Meta-Analyses (PRISMA) Guidelines for the Reporting of Meta-analyses eTable 2. Search Strategies on the PubMed eTable 3. Search Strategies on the Ovid Embase eTable 4. Search Strategies on the Cochrane Library eTable 5. List of References With Final Exclusion Reasons eFigure 1. Mean Difference of Improvement in VAS From Baseline to the End of Follow-up in the Laser Group eFigure 2. Mean Difference of Improvement in VAS From Baseline to the End of Follow-up in the Estrogen Group eFigure 3. Mean Difference of VHI From Baseline to the End of Follow-up in the Laser Group eFigure 4. Mean Difference of VHI From Baseline to the End of Follow-up in the Estrogen Group eFigure 5. Mean Difference of VMI From Baseline to the End of Follow-up in the Laser Group eFigure 6. Mean Difference of VMI From Baseline to the End of Follow-up in the Estrogen Group eFigure 7. Mean Difference of FSFI From Baseline to the End of Follow-up in the Laser Group eFigure 8. Mean Difference of FSFI From Baseline to the End of Follow-up in the Estrogen Group eFigure 9. Mean Difference of UDI-6 From Baseline to 6 Months in the Laser and Estrogen Group eFigure 10. Mean Difference Between the Laser and Estrogen Groups From Baseline to the End of Follow-up for ICIQ-UI SF eFigure 11. Mean Difference Between the Laser and Estrogen Groups From Baseline to the End of Follow-up for ICIQ-OAB [file jamanetwopen-e2232563-s001.pdf]

Supplementary Online Content

Jang YC, Leung CY, Huang HL. Comparison of severity of genitourinary syndrome of menopause symptoms after carbon dioxide laser vs vaginal estrogen therapy: a systematic review and meta-analysis. *JAMA Netw Open*. 2022;5(9):e2232563. doi:10.1001/jamanetworkopen.2022.32563

eTable 1. Preferred Reporting Items for Systematic Reviews and Meta-Analyses (PRISMA) Guidelines for the Reporting of Meta-analyses
eTable 2. Search Strategies on the PubMed
eTable 3. Search Strategies on the Ovid Embase
eTable 4. Search Strategies on the Cochrane Library
eTable 5. List of References With Final Exclusion Reasons
eFigure 1. Mean Difference of Improvement in VAS From Baseline to the End of Follow-up in the Laser Group
eFigure 2. Mean Difference of Improvement in VAS From Baseline to the End of Follow-up in the Estrogen Group
eFigure 3. Mean Difference of VHI From Baseline to the End of Follow-up in the Laser Group
eFigure 4. Mean Difference of VHI From Baseline to the End of Follow-up in the Estrogen Group
eFigure 5. Mean Difference of VMI From Baseline to the End of Follow-up in the Laser Group
eFigure 6. Mean Difference of VMI From Baseline to the End of Follow-up in the Estrogen Group
eFigure 7. Mean Difference of FSFI From Baseline to the End of Follow-up in the Laser Group
eFigure 8. Mean Difference of FSFI From Baseline to the End of Follow-up in the Estrogen Group
eFigure 9. Mean Difference of UDI-6 From Baseline to 6 Months in the Laser and Estrogen Group
eFigure 10. Mean Difference Between the Laser and Estrogen Groups From Baseline to the End of Follow-up for ICIQ-UI SF
eFigure 11. Mean Difference Between the Laser and Estrogen Groups From Baseline to the End of Follow-up for ICIQ-OAB

This supplementary material has been provided by the authors to give readers additional information about their work.

© 2022 Jang YC et al. *JAMA Network Open.*

eTable 1. Preferred Reporting Items for Systematic Reviews and Meta-Analyses (PRISMA) guidelines for the reporting of meta-analyses

Section and Topic	Item #	Checklist item	Location where item is reported
**TITLE**			
Title	1	Identify the report as a systematic review.	Title
**ABSTRACT**			
Abstract	2	See the PRISMA 2020 for Abstracts checklist.	Abstract
**INTRODUCTION**			
Rationale	3	Describe the rationale for the review in the context of existing knowledge.	Introduction, paragraph 1, 2
Objectives	4	Provide an explicit statement of the objective(s) or question(s) the review addresses.	Introduction, paragraph 3
**METHODS**			
Eligibility criteria	5	Specify the inclusion and exclusion criteria for the review and how studies were grouped for the syntheses.	Methods, paragraph 3
Information sources	6	Specify all databases, registers, websites, organisations, reference lists and other sources searched or consulted to identify studies. Specify the date when each source was last searched or consulted.	Methods, paragraph 2
Search strategy	7	Present the full search strategies for all databases, registers and websites, including any filters and limits used.	Supplement, eTable2, 3, 4
Selection process	8	Specify the methods used to decide whether a study met the inclusion criteria of the review, including how many reviewers screened each record and each report retrieved, whether they worked independently, and if applicable, details of automation tools used in the process.	Methods, paragraph 3
Data collection process	9	Specify the methods used to collect data from reports, including how many reviewers collected data from each report, whether they worked independently, any processes for obtaining or confirming data from study investigators, and if applicable, details of automation tools used in the process.	Methods, paragraph 5
Data items	10a	List and define all outcomes for which data were sought. Specify whether all results that were compatible with each outcome domain in each study were sought (e.g. for all measures, time points, analyses), and if not, the methods used to decide which results to collect.	Methods, paragraph 4
	10b	List and define all other variables for which data were sought (e.g. participant and intervention characteristics, funding sources). Describe any assumptions made about any missing or unclear information.	Methods, paragraph 5
Study risk of bias assessment	11	Specify the methods used to assess risk of bias in the included studies, including details of the tool(s) used, how many reviewers assessed each study and whether they worked independently, and if applicable, details of automation tools used in the process.	Methods, paragraph 5
Effect measures	12	Specify for each outcome the effect measure(s) (e.g. risk ratio, mean difference) used in the synthesis or presentation of results.	Methods, paragraph 6
Synthesis methods	13a	Describe the processes used to decide which studies were eligible for each synthesis (e.g. tabulating the study intervention characteristics and comparing against the planned groups for each synthesis (item #5)).	Methods, paragraph 5
	13b	Describe any methods required to prepare the data for	Methods,

Section and Topic	Item #	Checklist item	Location where item is reported
		presentation or synthesis, such as handling of missing summary statistics, or data conversions.	paragraph 6
	13c	Describe any methods used to tabulate or visually display results of individual studies and syntheses.	Methods, paragraph 6
	13d	Describe any methods used to synthesize results and provide a rationale for the choice(s). If meta-analysis was performed, describe the model(s), method(s) to identify the presence and extent of statistical heterogeneity, and software package(s) used.	Methods, paragraph 6
	13e	Describe any methods used to explore possible causes of heterogeneity among study results (e.g. subgroup analysis, meta-regression).	Methods, paragraph 6
	13f	Describe any sensitivity analyses conducted to assess robustness of the synthesized results.	N/A
Reporting bias assessment	14	Describe any methods used to assess risk of bias due to missing results in a synthesis (arising from reporting biases).	Methods, paragraph 5
Certainty assessment	15	Describe any methods used to assess certainty (or confidence) in the body of evidence for an outcome.	Methods, paragraph 6
**RESULTS**			
Study selection	16a	Describe the results of the search and selection process, from the number of records identified in the search to the number of studies included in the review, ideally using a flow diagram.	Results, paragraph 1. Figure 1
	16b	Cite studies that might appear to meet the inclusion criteria, but which were excluded, and explain why they were excluded.	Supplement, eTable 5
Study characteristics	17	Cite each included study and present its characteristics.	Results, paragraph 2. Table 1
Risk of bias in studies	18	Present assessments of risk of bias for each included study.	Results, paragraph 2. Figure 2
Results of individual studies	19	For all outcomes, present, for each study: (a) summary statistics for each group (where appropriate) and (b) an effect estimate and its precision (e.g. confidence/credible interval), ideally using structured tables or plots.	Figure 3. Supplement eFigure 1, 2, 3, 4, 5, 6, 7, 8
Results of syntheses	20a	For each synthesis, briefly summarise the characteristics and risk of bias among contributing studies.	Results, paragraph 3.
	20b	Present results of all statistical syntheses conducted. If meta-analysis was done, present for each the summary estimate and its precision (e.g. confidence/credible interval) and measures of statistical heterogeneity. If comparing groups, describe the direction of the effect.	Figure 3. Supplement eFigure 1, 2, 3, 4, 5, 6, 7, 8
	20c	Present results of all investigations of possible causes of heterogeneity among study results.	N/A
	20d	Present results of all sensitivity analyses conducted to assess the robustness of the synthesized results.	N/A
Reporting biases	21	Present assessments of risk of bias due to missing results (arising from reporting biases) for each synthesis assessed.	Figure 2
Certainty of	22	Present assessments of certainty (or confidence) in the body of	Figure 3. Supplement

© 2022 Jang YC et al. *JAMA Network Open.*

Section and Topic	Item #	Checklist item	Location where item is reported
evidence		evidence for each outcome assessed.	eFigure 1, 2, 3, 4, 5, 6, 7, 8
**DISCUSSION**			
Discussion	23a	Provide a general interpretation of the results in the context of other evidence.	Discussion, paragraph 1, 2, 3
	23b	Discuss any limitations of the evidence included in the review.	Discussion, paragraph 4
	23c	Discuss any limitations of the review processes used.	Discussion, paragraph 4
	23d	Discuss implications of the results for practice, policy, and future research.	Discussion, paragraph 5
**OTHER INFORMATION**			
Registration and protocol	24a	Provide registration information for the review, including register name and registration number, or state that the review was not registered.	Methods, paragraph 1
	24b	Indicate where the review protocol can be accessed, or state that a protocol was not prepared.	Methods, paragraph 1
	24c	Describe and explain any amendments to information provided at registration or in the protocol.	N/A
Support	25	Describe sources of financial or non-financial support for the review, and the role of the funders or sponsors in the review.	Funding/support. Role of funder/sponsor
Competing interests	26	Declare any competing interests of review authors.	Conflict of interest disclosure
Availability of data, code and other materials	27	Report which of the following are publicly available and where they can be found: template data collection forms; data extracted from included studies; data used for all analyses; analytic code; any other materials used in the review.	N/A

© 2022 Jang YC et al. *JAMA Network Open.*

eTable 2. Search strategies on the PubMed

#1 AND #2 AND #3 AND #4 (articles = 938)
#1 Intervention: CO2 laser therapy (articles = 356,999)<br>CO2 laser OR fractional CO2 laser OR laser OR carbon dioxide laser OR fractional laser OR microablative fractional CO2 laser therapy OR carbon dioxide laser OR vaginal laser therapy
#2 Control: Vaginal estrogen thearpy (articles = 10,778,902)<br>vaginal *estrogen OR *estrogen therapy OR *estrogen OR topical estradiol OR estradiol OR premarin vaginal cream OR promestriene
#3 Outcome: genitourinary syndrome of menopause (articles = 1,076,695)<br>Genitourinary syndrome of menopause OR postmenopausal OR genitourinary syndrome OR dyspareunia OR vaginal dryness OR pruritus OR irritation OR dysuria OR cell maturation index OR sexual function OR female sexual quotient OR sexual performance OR satisfaction OR visual analog scale OR urogenital distress inventory OR vaginal ph OR vaginal maturation index* OR maturation value of meisels OR female sexual function index OR Sexual Function Questionnaire Score OR vaginal health index OR international consultation on incontinence questionnaire OR stress urinary loss OR increased urinary frequency OR nocturia OR urgency OR urgency incontinence OR international consultation on incontinence questionnaire overactive bladder OR urinary symptoms
#3 Study Design: RCTs (articles = 1,067,308)<br>(randomized controlled trials OR clinical trial OR random) NOT (cohort studies OR longitudinal studies OR prospective studies OR retrospective studies OR survival analysis OR population-based stud* OR follow up studies OR follow-up OR cohort OR longitudinal OR prospective OR retrospective OR incidence stud* OR incidence stud* OR concurrent stud* OR follow up OR case control studies OR (case AND control) OR meta-analysis)

© 2022 Jang YC et al. *JAMA Network Open.*

eTable 3. Search strategies on the Ovid Embase

#1 AND #2 AND #3 AND #4 (articles = 17)
#1 Intervention: CO2 laser therapy (articles = 262,512)<br>CO2 laser OR fractional CO2 laser OR laser OR carbon dioxide laser OR fractional laser OR microablative fractional CO2 laser therapy OR carbon dioxide laser OR vaginal laser therapy
#2 Control: Vaginal estrogen thearpy (articles = 153,509)<br>vaginal *estrogen OR *estrogen therapy OR *estrogen OR topical estradiol OR estradiol OR premarin vaginal cream OR promestriene
#3 Outcome: genitourinary syndrome of menopause (articles = 475,293)<br>Genitourinary syndrome of menopause OR postmenopausal OR genitourinary syndrome OR dyspareunia OR vaginal dryness OR pruritus OR irritation OR dysuria OR cell maturation index OR sexual function OR female sexual quotient OR sexual performance OR satisfaction OR visual analog scale OR urogenital distress inventory OR vaginal ph OR vaginal maturation index* OR maturation value of meisels OR female sexual function index OR Sexual Function Questionnaire Score OR vaginal health index OR international consultation on incontinence questionnaire OR stress urinary loss OR increased urinary frequency OR nocturia OR urgency OR urgency incontinence OR international consultation on incontinence questionnaire overactive bladder OR urinary symptoms
#3 Study Design: RCTs (articles = 927,108)<br>(randomized controlled trials OR clinical trial OR random) NOT (cohort studies OR longitudinal studies OR prospective studies OR retrospective studies OR survival analysis OR population-based stud* OR follow up studies OR follow-up OR cohort OR longitudinal OR prospective OR retrospective OR incidence stud* OR incidence stud* OR concurrent stud* OR follow up OR case control studies OR (case AND control) OR meta-analysis)

© 2022 Jang YC et al. *JAMA Network Open.*

eTable 4. Search Strategies on the Cochrane Library

#1 AND #2 AND #3 AND #4 (articles = 0)
#1 Intervention: CO2 laser therapy (articles = 480)<br>CO2 laser OR fractional CO2 laser OR laser OR carbon dioxide laser OR fractional laser OR microablative fractional CO2 laser therapy OR carbon dioxide laser OR vaginal laser therapy
#2 Control: Vaginal estrogen thearpy (articles = 130)<br>vaginal *estrogen OR *estrogen therapy OR *estrogen OR topical estradiol OR estradiol OR premarin vaginal cream OR promestriene
#3 Outcome: genitourinary syndrome of menopause (articles = 2498)<br>Genitourinary syndrome of menopause OR postmenopausal OR genitourinary syndrome OR dyspareunia OR vaginal dryness OR pruritus OR irritation OR dysuria OR cell maturation index OR sexual function OR female sexual quotient OR sexual performance OR satisfaction OR visual analog scale OR urogenital distress inventory OR vaginal ph OR vaginal maturation index* OR maturation value of meisels OR female sexual function index OR Sexual Function Questionnaire Score OR vaginal health index OR international consultation on incontinence questionnaire OR stress urinary loss OR increased urinary frequency OR nocturia OR urgency OR urgency incontinence OR international consultation on incontinence questionnaire overactive bladder OR urinary symptoms
#3 Study Design: RCTs (articles = 3689)<br>(randomized controlled trials OR clinical trial OR random) NOT (cohort studies OR longitudinal studies OR prospective studies OR retrospective studies OR survival analysis OR population-based stud* OR follow up studies OR follow-up OR cohort OR longitudinal OR prospective OR retrospective OR incidence stud* OR incidence stud* OR concurrent stud* OR follow up OR case control studies OR (case AND control) OR meta-analysis)

© 2022 Jang YC et al. *JAMA Network Open.*

eTable 5. List of References with Final Exclusion Reasons

**Study**	**Reason of exclusion**
Mitchell CM, Reed SD, Diem S, Larson JC, Newton KM, Ensrud KE, LaCroix AZ, Caan B, Guthrie KA. Efficacy of vaginal estradiol or vaginal moisturizer vs placebo for treating postmenopausal vulvovaginal symptoms: a randomized clinical trial. *JAMA Internal Medicine* 2018; 178(5):681-90.	Inappropriate population
Cruff J, Khandwala S. A double-blind randomized sham-controlled trial to evaluate the efficacy of fractional carbon dioxide laser therapy on genitourinary syndrome of menopause. *The Journal of Sexual Medicine* 2021; 18(4):761-9.	Inappropriate population
Salvatore S, Pitsouni E, Grigoriadis T, Zacharakis D, Pantaleo G, Candiani M, Athanasiou S. CO2 laser and the genitourinary syndrome of menopause: a randomized sham-controlled trial. *Climacteric* 2021; *24*(2):187-193.	Inappropriate population
Quick AM, Dockter T, Le-Rademacher J, Salani R, Hudson C, Hundley A, Terstriep S, Streicher L, Faubion S, Loprinzi CL, Coleman JS. Pilot study of fractional CO2 laser therapy for genitourinary syndrome of menopause in gynecologic cancer survivors. *Maturitas* 2021; 144:37-44.	Inappropriate population
Ruanphoo P, Bunyavejchevin S. Treatment for vaginal atrophy using microablative fractional CO2 laser: a randomized double-blinded sham-controlled trial. *Menopause* 2020; 27(8):858-63.	Inappropriate population
Quick AM, Zvinovski F, Hudson C, Hundley A, Evans C, Suresh A, Stephens JA, Arthur E, Ramaswamy B, Reinbolt RE, Noonan AM. Fractional CO2 laser therapy for genitourinary syndrome of menopause for breast cancer survivors. *Supportive Care in Cancer* 2020; 28(8):3669-77.	Inappropriate population
Liu YY, Lin KL, Long CY. 'Long-term effects of vaginal erbium laser in the treatment of genitourinary syndrome of menopause': pelvic organ prolapse classification issues. *Climacteric* 2018; 21(5):515.	Observational study
Tovar-Huamani J, Mercado-Olivares F, Grandez-Urbina JA, Pichardo-Rodriguez R, Tovar-Huamani M, García-Perdomo H. Efficacy of fractional CO2 laser in the treatment of genitourinary syndrome of menopause in Latin-American population: First Peruvian experience. *Lasers in Surgery and Medicine* 2019; 51(6):509-15.	Observational study
Takacs P, Sipos AG, Kozma B, Cunningham TD, Larson K, Lampé R, Poka R. The effect of vaginal microablative fractional CO2 laser treatment on vaginal cytology. *Lasers in Surgery and Medicine* 2020; 52(8):708-12.	Observational study
Pitsouni E, Grigoriadis T, Tsiveleka A, Zacharakis D, Salvatore S, Athanasiou S. Microablative fractional CO2-laser therapy and the genitourinary syndrome of menopause: an observational study. *Maturitas* 2016; 94:131-6.	Observational study

© 2022 Jang YC et al. *JAMA Network Open.*

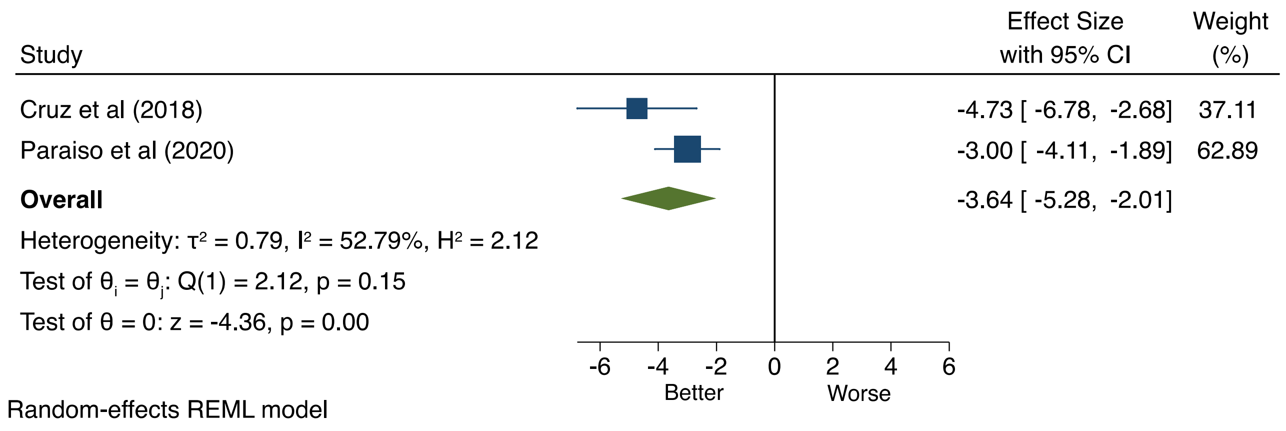

eFigure 1. Mean difference of improvement in VAS from baseline to the end of follow-up in the laser group
Abbreviation: CI, confidence intervals; REML, Restricted maximum likelihood; VAS, Vaginal Analog Scale

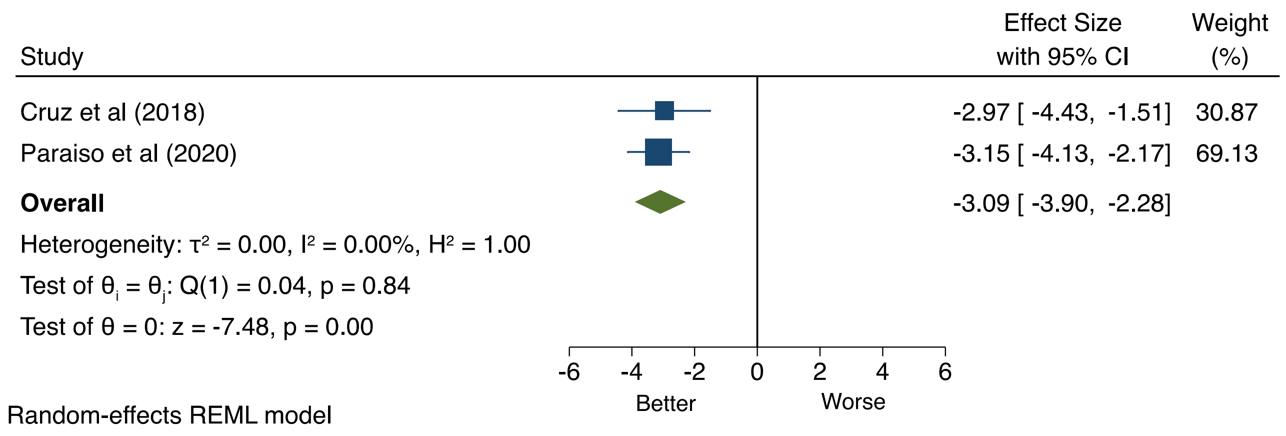

eFigure 2. Mean difference of improvement in VAS from baseline to the end of follow-up in the estrogen group
Abbreviation: CI, confidence intervals; REML, Restricted maximum likelihood; VAS, Vaginal Analog Scale

© 2022 Jang YC et al. *JAMA Network Open.*

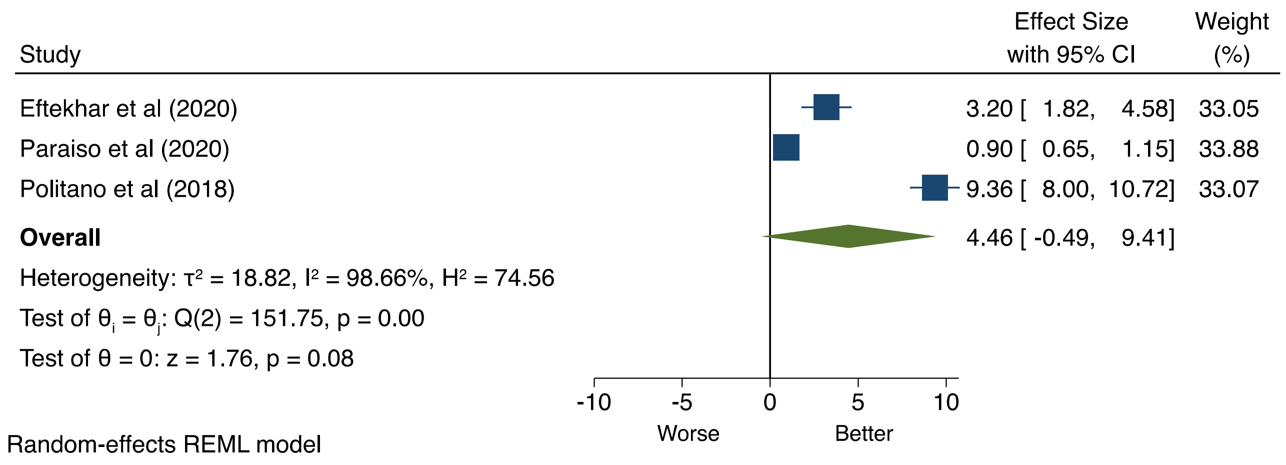

eFigure 3. Mean difference of VHI from baseline to the end of follow-up in the laser group
Abbreviation: CI, confidence intervals; REML, Restricted maximum likelihood; VHI, Vaginal Health Index

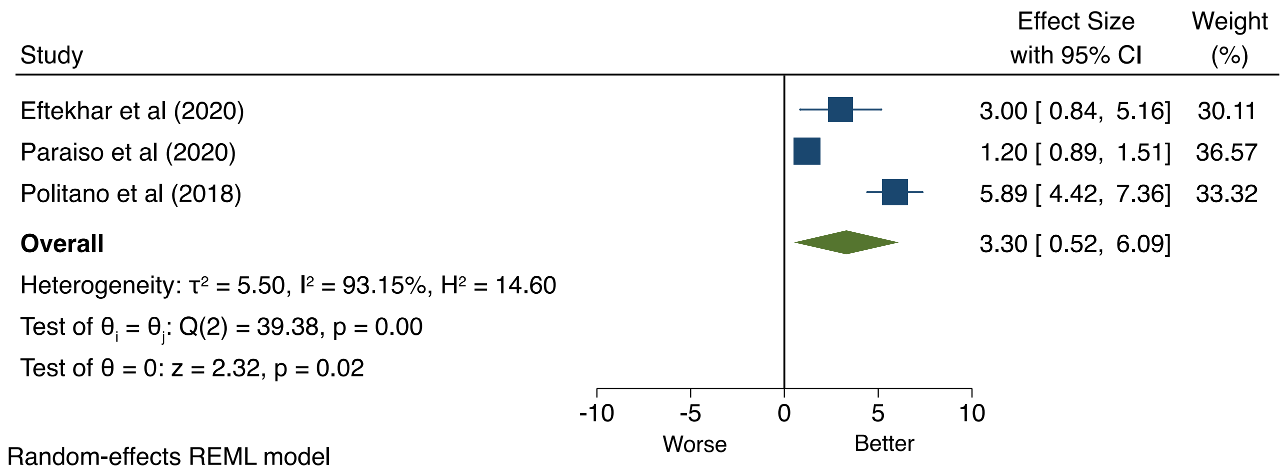

eFigure 4. Mean difference of VHI from baseline to the end of follow-up in the estrogen group
Abbreviation: CI, confidence intervals; REML, Restricted maximum likelihood; VHI, Vaginal Health Index

© 2022 Jang YC et al. *JAMA Network Open.*

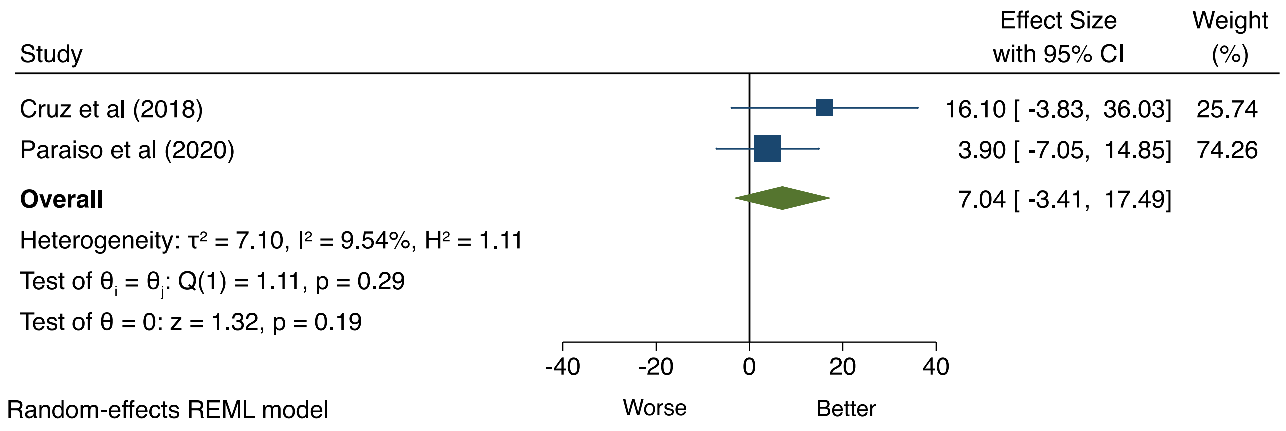

eFigure 5. Mean difference of VMI from baseline to the end of follow-up in the laser group
Abbreviation: CI, confidence intervals; REML, Restricted maximum likelihood; VMI, Vaginal Maturation Index

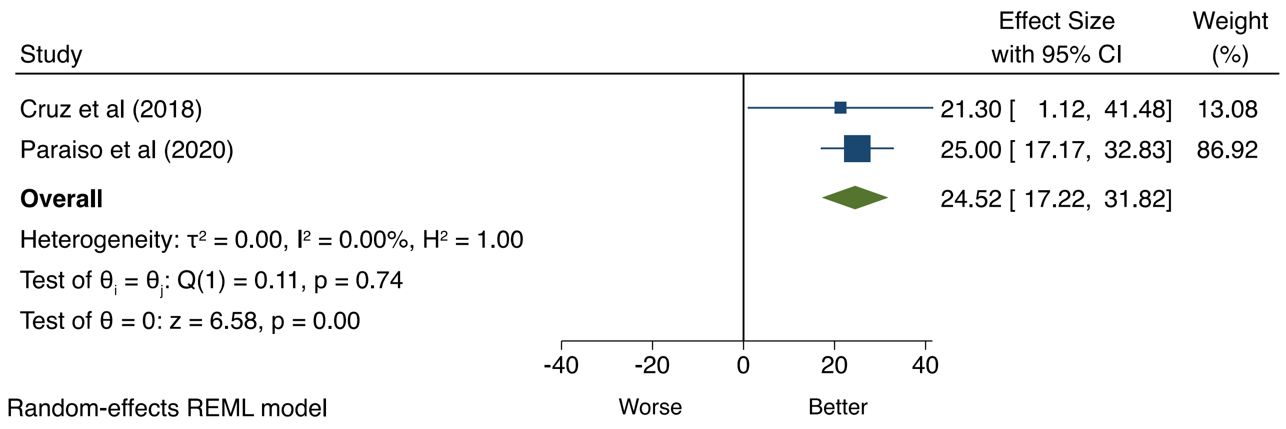

eFigure 6. Mean difference of VMI from baseline to the end of follow-up in the estrogen group
Abbreviation: CI, confidence intervals; REML, Restricted maximum likelihood; VMI, Vaginal Maturation Index

© 2022 Jang YC et al. *JAMA Network Open.*

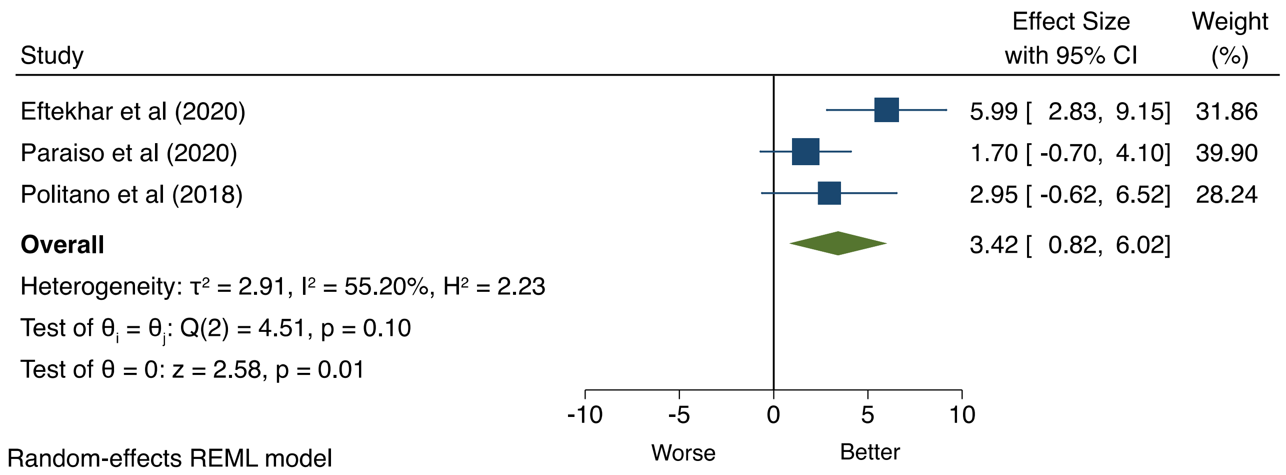

eFigure 7. Mean difference of FSFI from baseline to the end of follow-up in the laser group
Abbreviation: CI, confidence intervals; REML, Restricted maximum likelihood; FSFI, Female Sexual Function Index.

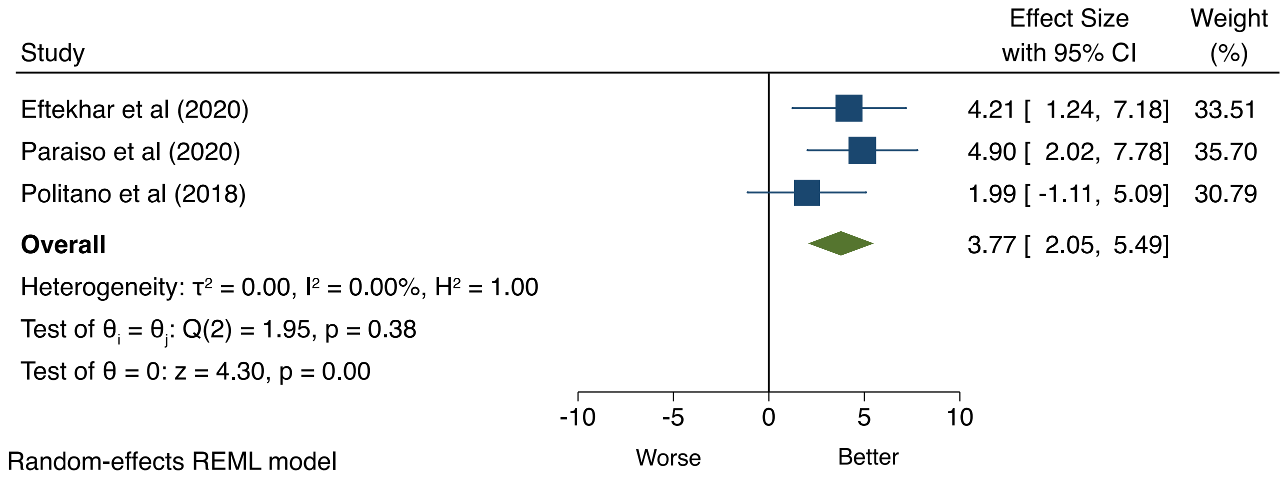

eFigure 8. Mean difference of FSFI from baseline to the end of follow-up in the estrogen group
Abbreviation: CI, confidence intervals; REML, Restricted maximum likelihood; FSFI, Female Sexual Function Index.

© 2022 Jang YC et al. *JAMA Network Open.*

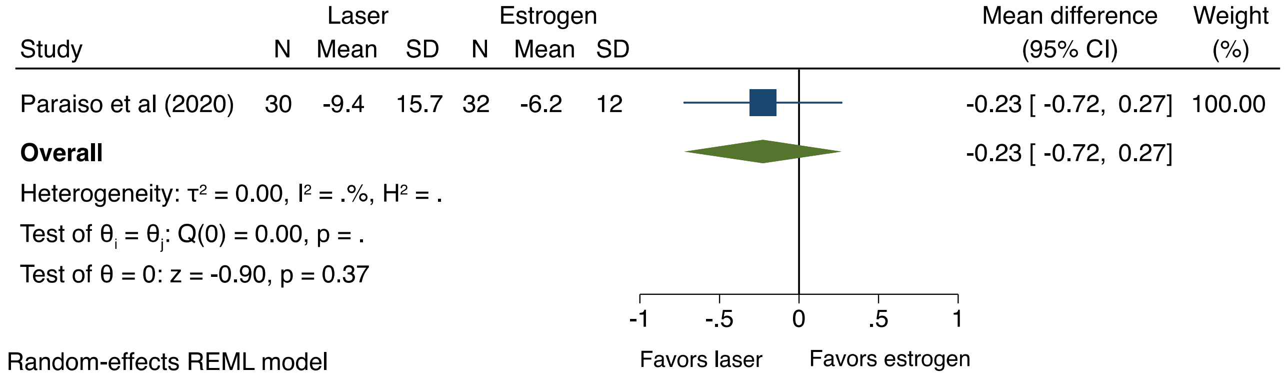

eFigure 9. Mean difference of UDI-6 from baseline to 6 months in the laser and estrogen group
Abbreviation: CI, confidence intervals; REML, Restricted maximum likelihood; UDI-6, Urogenital Distress Inventory score

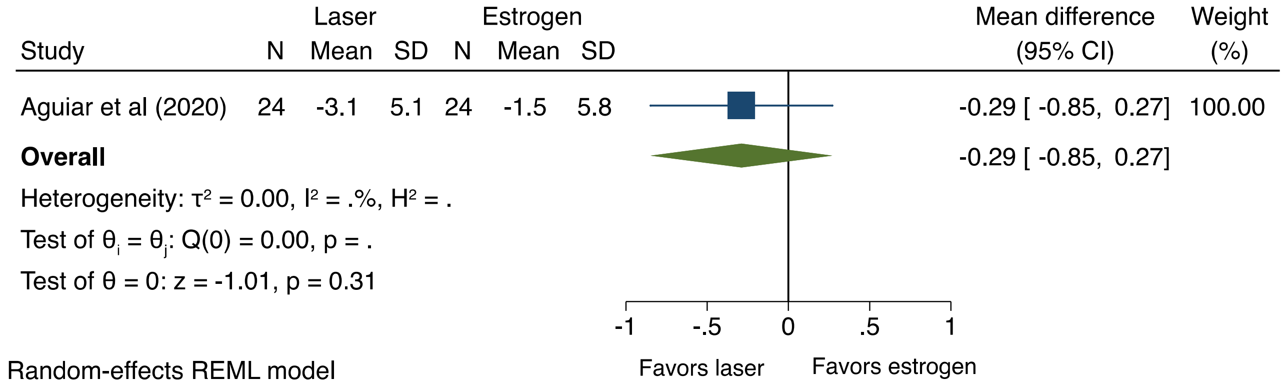

eFigure 10. Mean difference between the laser and estrogen groups from baseline to the end of follow up for ICIQ-UI SF
Abbreviation: CI, confidence intervals; REML, Restricted maximum likelihood; ICIQ-UI SF, International Consultation on Incontinence Questionnaire-Short Form

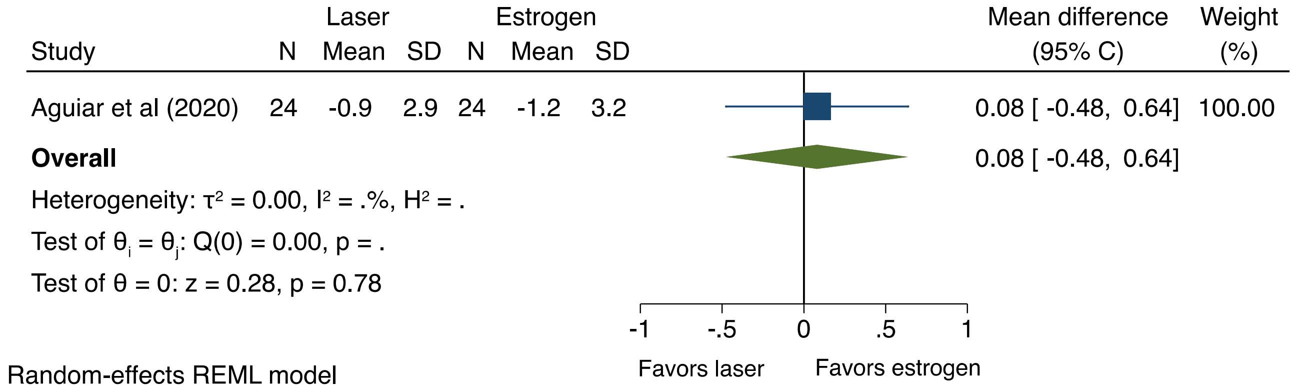

eFigure 11. Mean difference between the laser and estrogen groups from baseline to the end of follow up for ICIQ-OAB
Abbreviation: CI, confidence intervals; REML, Restricted maximum likelihood; ICIQ-OAB, International Consultation on Incontinence Questionnaire Overactive Bladder

© 2022 Jang YC et al. *JAMA Network Open.*
